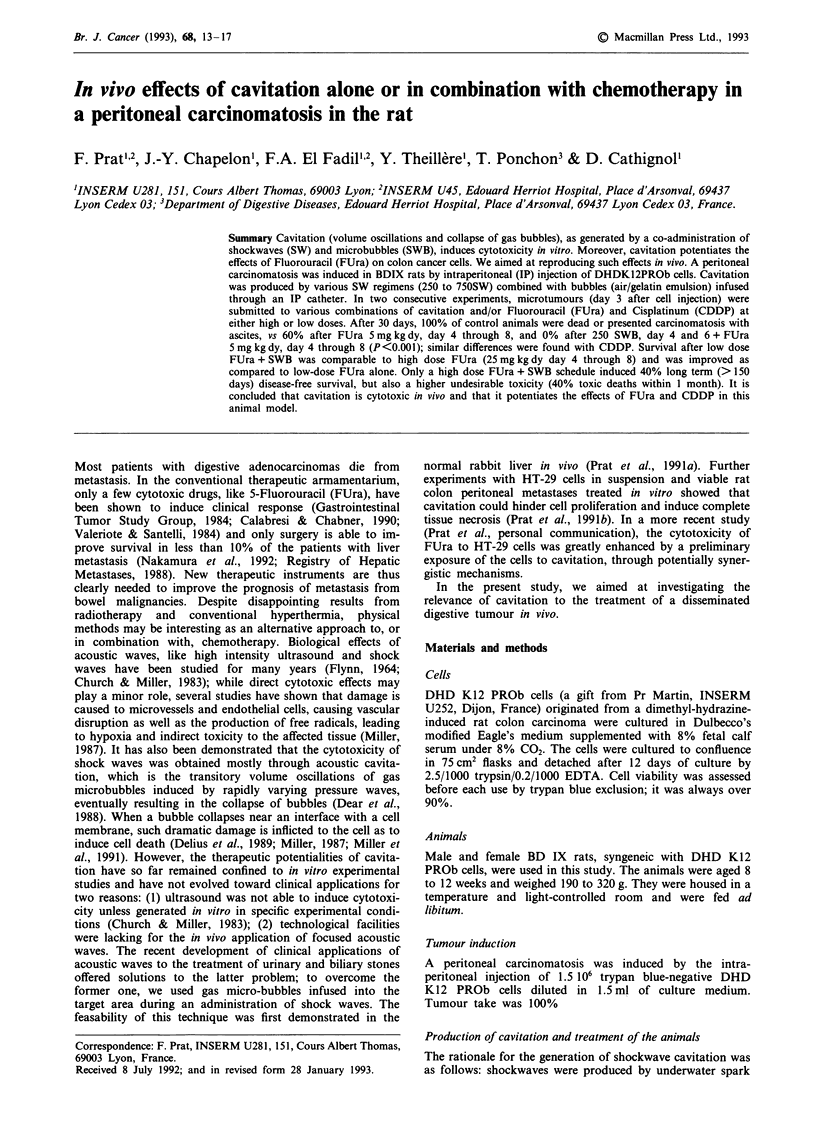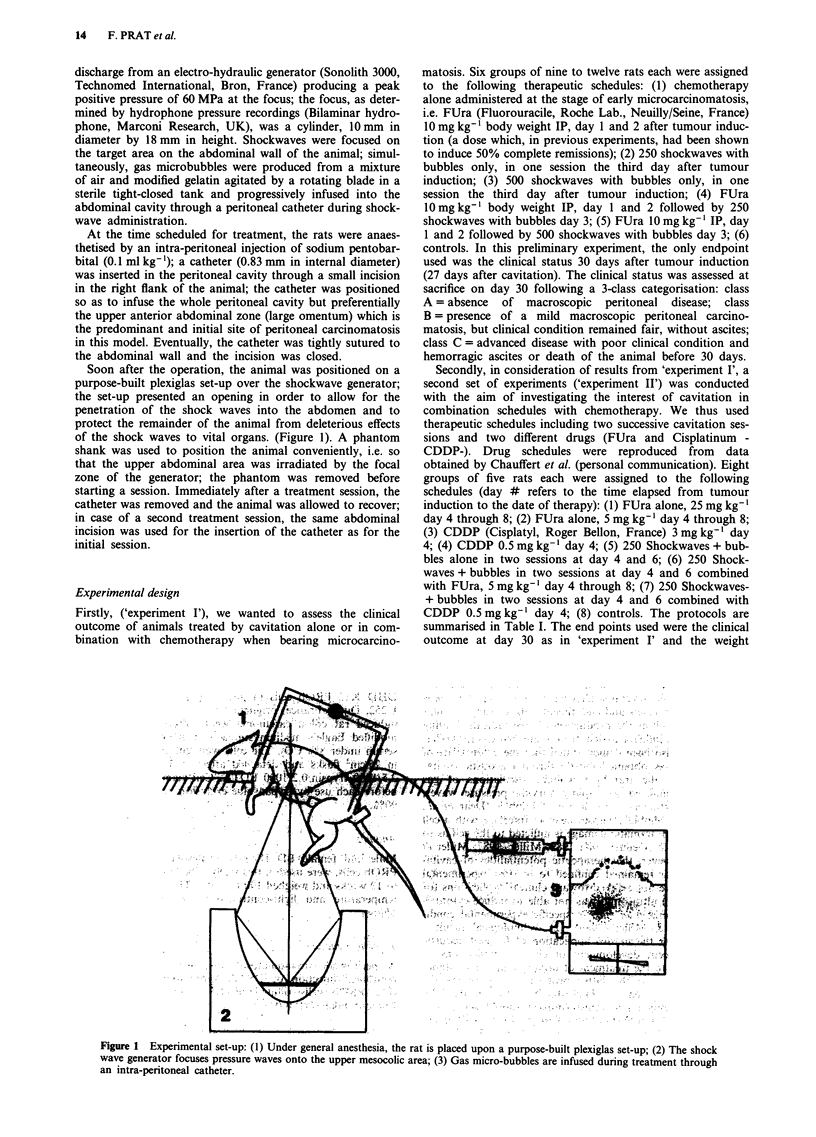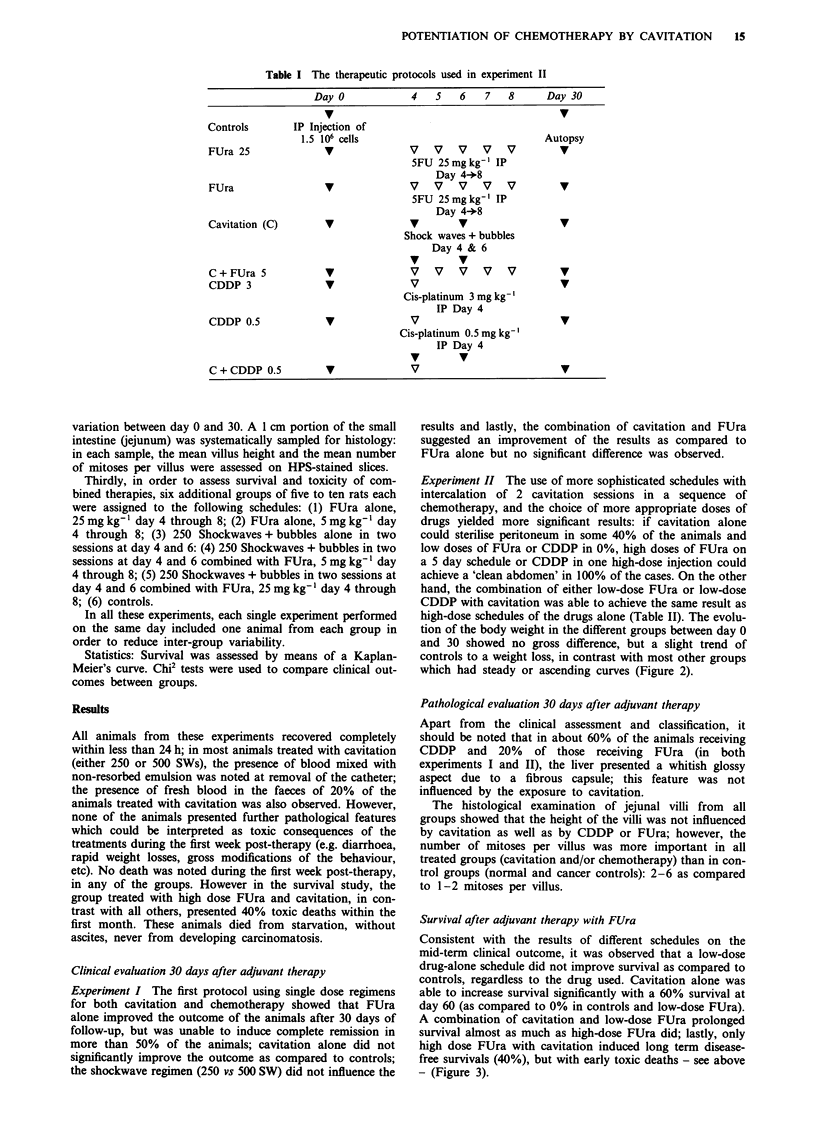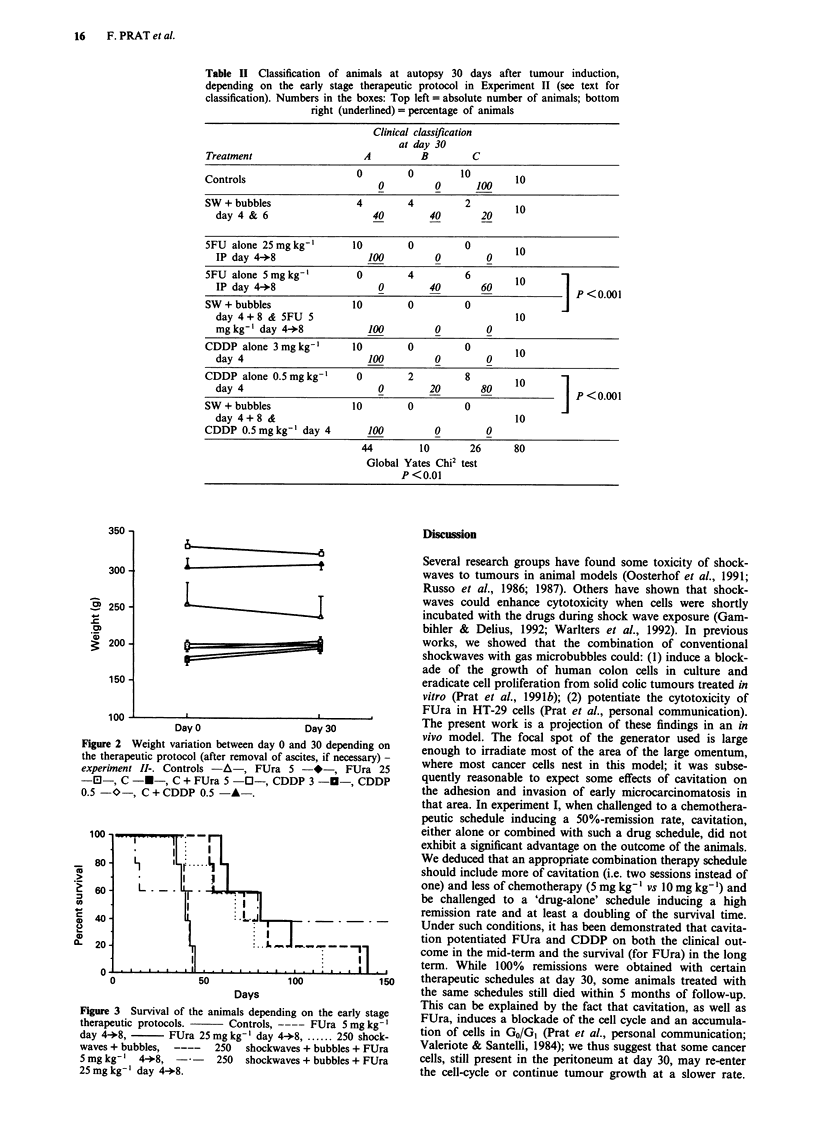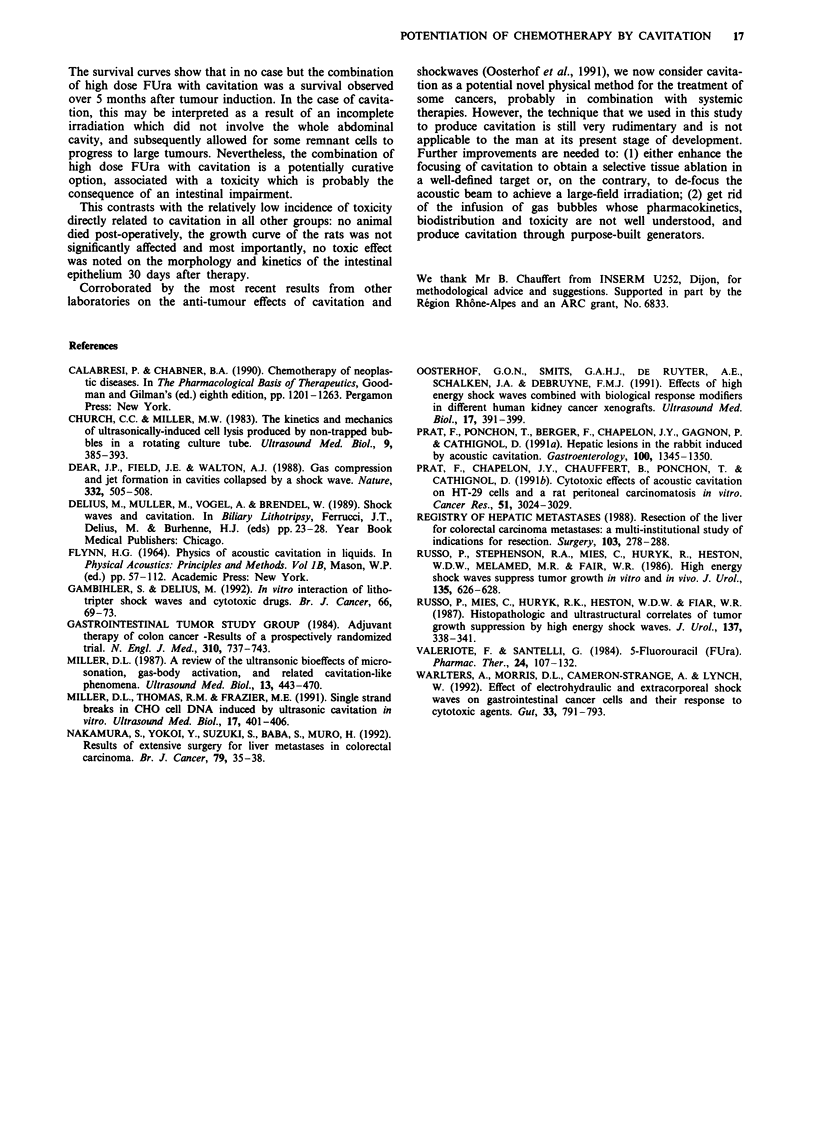# In vivo effects of cavitation alone or in combination with chemotherapy in a peritoneal carcinomatosis in the rat.

**DOI:** 10.1038/bjc.1993.279

**Published:** 1993-07

**Authors:** F. Prat, J. Y. Chapelon, F. A. el Fadil, Y. Theillère, T. Ponchon, D. Cathignol

**Affiliations:** INSERM U281, Lyon, France.

## Abstract

Cavitation (volume oscillations and collapse of gas bubbles), as generated by a co-administration of shockwaves (SW) and microbubbles (SWB), induces cytotoxicity in vitro. Moreover, cavitation potentiates the effects of Fluorouracil (FUra) on colon cancer cells. We aimed at reproducing such effects in vivo. A peritoneal carcinomatosis was induced in BDIX rats by intraperitoneal (IP) injection of DHDK12PROb cells. Cavitation was produced by various SW regimens (250 to 750SW) combined with bubbles (air/gelatin emulsion) infused through an IP catheter. In two consecutive experiments, microtumours (day 3 after cell injection) were submitted to various combinations of cavitation and/or Fluorouracil (FUra) and Cisplatinum (CDDP) at either high or low doses. After 30 days, 100% of control animals were dead or presented carcinomatosis with ascites, vs 60% after FUra 5 mg kg dy, day 4 through 8, and 0% after 250 SWB, day 4 and 6 + FUra 5 mg kg dy, day 4 through 8 (P < 0.001); similar differences were found with CDDP. Survival after low dose FUra + SWB was comparable to high dose FUra (25 mg kg dy day through 8) and was improved as compared to low-dose FUra alone. Only a high dose FUra + SWB schedule induced 40% long term (> 150 days) disease-free survival, but also a higher undesirable toxicity (40% toxic deaths within 1 month). It is concluded that cavitation is cytotoxic in vivo and that it potentiates the effects of FUra and CDDP in this animal model.


Br. J. Cancer (1993), 68, 13–17 © Macmillan Press Ltd., 1993

### *In vivo* effects of cavitation alone or in combination with chemotherapy in a peritoneal carcinomatosis in the rat

F. Prat[1,2], J.-Y. Chapelon[1], F.A. El Fadil[1,2], Y. Theillère[1], T. Ponchon[3] & D. Cathignol[1]

[1]*INSERM U281, 151, Cours Albert Thomas, 69003 Lyon;* [2]*INSERM U45, Edouard Herriot Hospital, Place d'Arsonval, 69437 Lyon Cedex 03;* [3]*Department of Digestive Diseases, Edouard Herriot Hospital, Place d'Arsonval, 69437 Lyon Cedex 03, France.*

**Summary** Cavitation (volume oscillations and collapse of gas bubbles), as generated by a co-administration of shockwaves (SW) and microbubbles (SWB), induces cytotoxicity *in vitro*. Moreover, cavitation potentiates the effects of Fluorouracil (FUra) on colon cancer cells. We aimed at reproducing such effects *in vivo*. A peritoneal carcinomatosis was induced in BDIX rats by intraperitoneal (IP) injection of DHDK12PROb cells. Cavitation was produced by various SW regimens (250 to 750SW) combined with bubbles (air/gelatin emulsion) infused through an IP catheter. In two consecutive experiments, microtumours (day 3 after cell injection) were submitted to various combinations of cavitation and/or Fluorouracil (FUra) and Cisplatinum (CDDP) at either high or low doses. After 30 days, 100% of control animals were dead or presented carcinomatosis with ascites, *vs* 60% after FUra 5 mg kg dy, day 4 through 8, and 0% after 250 SWB, day 4 and 6 + FUra 5 mg kg dy, day 4 through 8 ($P < 0.001$); similar differences were found with CDDP. Survival after low dose FUra + SWB was comparable to high dose FUra (25 mg kg dy day 4 through 8) and was improved as compared to low-dose FUra alone. Only a high dose FUra + SWB schedule induced 40% long term (> 150 days) disease-free survival, but also a higher undesirable toxicity (40% toxic deaths within 1 month). It is concluded that cavitation is cytotoxic *in vivo* and that it potentiates the effects of FUra and CDDP in this animal model.

Most patients with digestive adenocarcinomas die from metastasis. In the conventional therapeutic armamentarium, only a few cytotoxic drugs, like 5-Fluorouracil (FUra), have been shown to induce clinical response (Gastrointestinal Tumor Study Group, 1984; Calabresi & Chabner, 1990; Valeriote & Santelli, 1984) and only surgery is able to improve survival in less than 10% of the patients with liver metastasis (Nakamura *et al*., 1992; Registry of Hepatic Metastases, 1988). New therapeutic instruments are thus clearly needed to improve the prognosis of metastasis from bowel malignancies. Despite disappointing results from radiotherapy and conventional hyperthermia, physical methods may be interesting as an alternative approach to, or in combination with, chemotherapy. Biological effects of acoustic waves, like high intensity ultrasound and shock waves have been studied for many years (Flynn, 1964; Church & Miller, 1983); while direct cytotoxic effects may play a minor role, several studies have shown that damage is caused to microvessels and endothelial cells, causing vascular disruption as well as the production of free radicals, leading to hypoxia and indirect toxicity to the affected tissue (Miller, 1987). It has also been demonstrated that the cytotoxicity of shock waves was obtained mostly through acoustic cavitation, which is the transitory volume oscillations of gas microbubbles induced by rapidly varying pressure waves, eventually resulting in the collapse of bubbles (Dear *et al*., 1988). When a bubble collapses near an interface with a cell membrane, such dramatic damage is inflicted to the cell as to induce cell death (Delius *et al*., 1989; Miller, 1987; Miller *et al*., 1991). However, the therapeutic potentialities of cavitation have so far remained confined to *in vitro* experimental studies and have not evolved toward clinical applications for two reasons: (1) ultrasound was not able to induce cytotoxicity unless generated *in vitro* in specific experimental conditions (Church & Miller, 1983); (2) technological facilities were lacking for the *in vivo* application of focused acoustic waves. The recent development of clinical applications of acoustic waves to the treatment of urinary and biliary stones offered solutions to the latter problem; to overcome the former one, we used gas micro-bubbles infused into the target area during an administration of shock waves. The feasability of this technique was first demonstrated in the normal rabbit liver *in vivo* (Prat *et al*., 1991*a*). Further experiments with HT-29 cells in suspension and viable rat colon peritoneal metastases treated *in vitro* showed that cavitation could hinder cell proliferation and induce complete tissue necrosis (Prat *et al*., 1991*b*). In a more recent study (Prat *et al*., personal communication), the cytotoxicity of FUra to HT-29 cells was greatly enhanced by a preliminary exposure of the cells to cavitation, through potentially synergistic mechanisms.

In the present study, we aimed at investigating the relevance of cavitation to the treatment of a disseminated digestive tumour *in vivo*.

Correspondence: F. Prat, INSERM U281, 151, Cours Albert Thomas, 69003 Lyon, France.
Received 8 July 1992; and in revised form 28 January 1993.

### Materials and methods

### *Cells*

DHD K12 PROb cells (a gift from Pr Martin, INSERM U252, Dijon, France) originated from a dimethyl-hydrazine-induced rat colon carcinoma were cultured in Dulbecco's modified Eagle's medium supplemented with 8% fetal calf serum under 8% $CO_2$. The cells were cultured to confluence in 75 $cm^2$ flasks and detached after 12 days of culture by 2.5/1000 trypsin/0.2/1000 EDTA. Cell viability was assessed before each use by trypan blue exclusion; it was always over 90%.

### *Animals*

Male and female BD IX rats, syngeneic with DHD K12 PROb cells, were used in this study. The animals were aged 8 to 12 weeks and weighed 190 to 320 g. They were housed in a temperature and light-controlled room and were fed *ad libitum*.

### *Tumour induction*

A peritoneal carcinomatosis was induced by the intraperitoneal injection of 1.5 $10^6$ trypan blue-negative DHD K12 PROb cells diluted in 1.5 ml of culture medium. Tumour take was 100%

### *Production of cavitation and treatment of the animals*

The rationale for the generation of shockwave cavitation was as follows: shockwaves were produced by underwater spark discharge from an electro-hydraulic generator (Sonolith 3000, Technomed International, Bron, France) producing a peak positive pressure of 60 MPa at the focus; the focus, as determined by hydrophone pressure recordings (Bilaminar hydrophone, Marconi Research, UK), was a cylinder, 10 mm in diameter by 18 mm in height. Shockwaves were focused on the target area on the abdominal wall of the animal; simultaneously, gas microbubbles were produced from a mixture of air and modified gelatin agitated by a rotating blade in a sterile tight-closed tank and progressively infused into the abdominal cavity through a peritoneal catheter during shockwave administration.

At the time scheduled for treatment, the rats were anaesthetised by an intra-peritoneal injection of sodium pentobarbital (0.1 ml $kg^{-1}$); a catheter (0.83 mm in internal diameter) was inserted in the peritoneal cavity through a small incision in the right flank of the animal; the catheter was positioned so as to infuse the whole peritoneal cavity but preferentially the upper anterior abdominal zone (large omentum) which is the predominant and initial site of peritoneal carcinomatosis in this model. Eventually, the catheter was tightly sutured to the abdominal wall and the incision was closed.

Soon after the operation, the animal was positioned on a purpose-built plexiglas set-up over the shockwave generator; the set-up presented an opening in order to allow for the penetration of the shock waves into the abdomen and to protect the remainder of the animal from deleterious effects of the shock waves to vital organs. (Figure 1). A phantom shank was used to position the animal conveniently, i.e. so that the upper abdominal area was irradiated by the focal zone of the generator; the phantom was removed before starting a session. Immediately after a treatment session, the catheter was removed and the animal was allowed to recover; in case of a second treatment session, the same abdominal incision was used for the insertion of the catheter as for the initial session.

### Experimental design

Firstly, ('experiment I'), we wanted to assess the clinical outcome of animals treated by cavitation alone or in combination with chemotherapy when bearing microcarcinomatosis. Six groups of nine to twelve rats each were assigned to the following therapeutic schedules: (1) chemotherapy alone administered at the stage of early microcarcinomatosis, i.e. FUra (Fluorouracile, Roche Lab., Neuilly/Seine, France) 10 mg $kg^{-1}$ body weight IP, day 1 and 2 after tumour induction (a dose which, in previous experiments, had been shown to induce 50% complete remissions); (2) 250 shockwaves with bubbles only, in one session the third day after tumour induction; (3) 500 shockwaves with bubbles only, in one session the third day after tumour induction; (4) FUra 10 mg $kg^{-1}$ body weight IP, day 1 and 2 followed by 250 shockwaves with bubbles day 3; (5) FUra 10 mg $kg^{-1}$ IP, day 1 and 2 followed by 500 shockwaves with bubbles day 3; (6) controls. In this preliminary experiment, the only endpoint used was the clinical status 30 days after tumour induction (27 days after cavitation). The clinical status was assessed at sacrifice on day 30 following a 3-class categorisation: class A = absence of macroscopic peritoneal disease; class B = presence of a mild macroscopic peritoneal carcinomatosis, but clinical condition remained fair, without ascites; class C = advanced disease with poor clinical condition and hemorragic ascites or death of the animal before 30 days.

Secondly, in consideration of results from 'experiment I', a second set of experiments ('experiment II') was conducted with the aim of investigating the interest of cavitation in combination schedules with chemotherapy. We thus used therapeutic schedules including two successive cavitation sessions and two different drugs (FUra and Cisplatinum - CDDP-). Drug schedules were reproduced from data obtained by Chauffert et al. (personal communication). Eight groups of five rats each were assigned to the following schedules (day # refers to the time elapsed from tumour induction to the date of therapy): (1) FUra alone, 25 mg $kg^{-1}$ day 4 through 8; (2) FUra alone, 5 mg $kg^{-1}$ day 4 through 8; (3) CDDP (Cisplatyl, Roger Bellon, France) 3 mg $kg^{-1}$ day 4; (4) CDDP 0.5 mg $kg^{-1}$ day 4; (5) 250 Shockwaves + bubbles alone in two sessions at day 4 and 6; (6) 250 Shockwaves + bubbles in two sessions at day 4 and 6 combined with FUra, 5 mg $kg^{-1}$ day 4 through 8; (7) 250 Shockwaves- + bubbles in two sessions at day 4 and 6 combined with CDDP 0.5 mg $kg^{-1}$ day 4; (8) controls. The protocols are summarised in Table I. The end points used were the clinical outcome at day 30 as in 'experiment I' and the weight

**Figure 1** Experimental set-up: (1) Under general anesthesia, the rat is placed upon a purpose-built plexiglas set-up; (2) The shock wave generator focuses pressure waves onto the upper mesocolic area; (3) Gas micro-bubbles are infused during treatment through an intra-peritoneal catheter.

**Table I** The therapeutic protocols used in experiment II

| | *Day 0* | *4* | *5* | *6* | *7* | *8* | *Day 30* |
|---|---|---|---|---|---|---|---|
| | ▼ | | | | | | ▼ |
| Controls | IP Injection of 1.5 $10^6$ cells | | | | | | Autopsy |
| FUra 25 | ▼ | ▽ | ▽ | ▽ | ▽ | ▽ | ▼ |
| | | 5FU 25 mg $kg^{-1}$ IP Day 4→8 | | | | | |
| FUra | ▼ | ▽ | ▽ | ▽ | ▽ | ▽ | ▼ |
| | | 5FU 25 mg $kg^{-1}$ IP Day 4→8 | | | | | |
| Cavitation (C) | ▼ | ▼ | | ▼ | | | ▼ |
| | | Shock waves + bubbles Day 4 & 6 | | | | | |
| | | ▼ | | ▼ | | | |
| C + FUra 5 | ▼ | ▽ | ▽ | ▽ | ▽ | ▽ | ▼ |
| CDDP 3 | ▼ | ▽ | | | | | ▼ |
| | | Cis-platinum 3 mg $kg^{-1}$ IP Day 4 | | | | | |
| CDDP 0.5 | ▼ | ▽ | | | | | ▼ |
| | | Cis-platinum 0.5 mg $kg^{-1}$ IP Day 4 | | | | | |
| | | ▼ | | ▼ | | | |
| C + CDDP 0.5 | ▼ | ▽ | | | | | ▼ |

variation between day 0 and 30. A 1 cm portion of the small intestine (jejunum) was systematically sampled for histology: in each sample, the mean villus height and the mean number of mitoses per villus were assessed on HPS-stained slices.

Thirdly, in order to assess survival and toxicity of combined therapies, six additional groups of five to ten rats each were assigned to the following schedules: (1) FUra alone, 25 mg $kg^{-1}$ day 4 through 8; (2) FUra alone, 5 mg $kg^{-1}$ day 4 through 8; (3) 250 Shockwaves + bubbles alone in two sessions at day 4 and 6: (4) 250 Shockwaves + bubbles in two sessions at day 4 and 6 combined with FUra, 5 mg $kg^{-1}$ day 4 through 8; (5) 250 Shockwaves + bubbles in two sessions at day 4 and 6 combined with FUra, 25 mg $kg^{-1}$ day 4 through 8; (6) controls.

In all these experiments, each single experiment performed on the same day included one animal from each group in order to reduce inter-group variability.

Statistics: Survival was assessed by means of a Kaplan-Meier's curve. $Chi^2$ tests were used to compare clinical outcomes between groups.

### Results

All animals from these experiments recovered completely within less than 24 h; in most animals treated with cavitation (either 250 or 500 SWs), the presence of blood mixed with non-resorbed emulsion was noted at removal of the catheter; the presence of fresh blood in the faeces of 20% of the animals treated with cavitation was also observed. However, none of the animals presented further pathological features which could be interpreted as toxic consequences of the treatments during the first week post-therapy (e.g. diarrhoea, rapid weight losses, gross modifications of the behaviour, etc). No death was noted during the first week post-therapy, in any of the groups. However in the survival study, the group treated with high dose FUra and cavitation, in contrast with all others, presented 40% toxic deaths within the first month. These animals died from starvation, without ascites, never from developing carcinomatosis.

### *Clinical evaluation 30 days after adjuvant therapy*

*Experiment I* The first protocol using single dose regimens for both cavitation and chemotherapy showed that FUra alone improved the outcome of the animals after 30 days of follow-up, but was unable to induce complete remission in more than 50% of the animals; cavitation alone did not significantly improve the outcome as compared to controls; the shockwave regimen (250 *vs* 500 SW) did not influence the results and lastly, the combination of cavitation and FUra suggested an improvement of the results as compared to FUra alone but no significant difference was observed.

*Experiment II* The use of more sophisticated schedules with intercalation of 2 cavitation sessions in a sequence of chemotherapy, and the choice of more appropriate doses of drugs yielded more significant results: if cavitation alone could sterilise peritoneum in some 40% of the animals and low doses of FUra or CDDP in 0%, high doses of FUra on a 5 day schedule or CDDP in one high-dose injection could achieve a 'clean abdomen' in 100% of the cases. On the other hand, the combination of either low-dose FUra or low-dose CDDP with cavitation was able to achieve the same result as high-dose schedules of the drugs alone (Table II). The evolution of the body weight in the different groups between day 0 and 30 showed no gross difference, but a slight trend of controls to a weight loss, in contrast with most other groups which had steady or ascending curves (Figure 2).

### *Pathological evaluation 30 days after adjuvant therapy*

Apart from the clinical assessment and classification, it should be noted that in about 60% of the animals receiving CDDP and 20% of those receiving FUra (in both experiments I and II), the liver presented a whitish glossy aspect due to a fibrous capsule; this feature was not influenced by the exposure to cavitation.

The histological examination of jejunal villi from all groups showed that the height of the villi was not influenced by cavitation as well as by CDDP or FUra; however, the number of mitoses per villus was more important in all treated groups (cavitation and/or chemotherapy) than in control groups (normal and cancer controls): 2–6 as compared to 1–2 mitoses per villus.

### *Survival after adjuvant therapy with FUra*

Consistent with the results of different schedules on the mid-term clinical outcome, it was observed that a low-dose drug-alone schedule did not improve survival as compared to controls, regardless to the drug used. Cavitation alone was able to increase survival significantly with a 60% survival at day 60 (as compared to 0% in controls and low-dose FUra). A combination of cavitation and low-dose FUra prolonged survival almost as much as high-dose FUra did; lastly, only high dose FUra with cavitation induced long term disease-free survivals (40%), but with early toxic deaths – see above – (Figure 3).

**Table II** Classification of animals at autopsy 30 days after tumour induction, depending on the early stage therapeutic protocol in Experiment II (see text for classification). Numbers in the boxes: Top left = absolute number of animals; bottom right (underlined) = percentage of animals

| *Treatment* | *Clinical classification at day 30* *A* | *B* | *C* | | |
|---|---|---|---|---|---|
| Controls | 0 / 0 | 0 / 0 | 10 / 100 | 10 | |
| SW + bubbles day 4 & 6 | 4 / 40 | 4 / 40 | 2 / 20 | 10 | |
| 5FU alone 25 mg kg$^{-1}$ IP day 4→8 | 10 / 100 | 0 / 0 | 0 / 0 | 10 | |
| 5FU alone 5 mg kg$^{-1}$ IP day 4→8 | 0 / 0 | 4 / 40 | 6 / 60 | 10 | $P < 0.001$ |
| SW + bubbles day 4 + 8 & 5FU 5 mg kg$^{-1}$ day 4→8 | 10 / 100 | 0 / 0 | 0 / 0 | 10 | |
| CDDP alone 3 mg kg$^{-1}$ day 4 | 10 / 100 | 0 / 0 | 0 / 0 | 10 | |
| CDDP alone 0.5 mg kg$^{-1}$ day 4 | 0 / 0 | 2 / 20 | 8 / 80 | 10 | $P < 0.001$ |
| SW + bubbles day 4 + 8 & CDDP 0.5 mg kg$^{-1}$ day 4 | 10 / 100 | 0 / 0 | 0 / 0 | 10 | |
| | 44 | 10 | 26 | 80 | |
| | Global Yates Chi$^2$ test $P < 0.01$ | | | | |

**Figure 2** Weight variation between day 0 and 30 depending on the therapeutic protocol (after removal of ascites, if necessary) – *experiment II-*. Controls —Δ—, FUra 5 —◆—, FUra 25 —□—, C —■—, C + FUra 5 —□—, CDDP 3 —■—, CDDP 0.5 —◇—, C + CDDP 0.5 —▲—.

**Figure 3** Survival of the animals depending on the early stage therapeutic protocols. ——— Controls, ---- FUra 5 mg kg$^{-1}$ day 4→8, ——— FUra 25 mg kg$^{-1}$ day 4→8, ...... 250 shockwaves + bubbles, ---- 250 shockwaves + bubbles + FUra 5 mg kg$^{-1}$ 4→8, —·— 250 shockwaves + bubbles + FUra 25 mg kg$^{-1}$ day 4→8.

### Discussion

Several research groups have found some toxicity of shockwaves to tumours in animal models (Oosterhof *et al.*, 1991; Russo *et al.*, 1986; 1987). Others have shown that shockwaves could enhance cytotoxicity when cells were shortly incubated with the drugs during shock wave exposure (Gambihler & Delius, 1992; Warlters *et al.*, 1992). In previous works, we showed that the combination of conventional shockwaves with gas microbubbles could: (1) induce a blockade of the growth of human colon cells in culture and eradicate cell proliferation from solid colic tumours treated *in vitro* (Prat *et al.*, 1991*b*); (2) potentiate the cytotoxicity of FUra in HT-29 cells (Prat *et al.*, personal communication). The present work is a projection of these findings in an *in vivo* model. The focal spot of the generator used is large enough to irradiate most of the area of the large omentum, where most cancer cells nest in this model; it was subsequently reasonable to expect some effects of cavitation on the adhesion and invasion of early microcarcinomatosis in that area. In experiment I, when challenged to a chemotherapeutic schedule inducing a 50%-remission rate, cavitation, either alone or combined with such a drug schedule, did not exhibit a significant advantage on the outcome of the animals. We deduced that an appropriate combination therapy schedule should include more of cavitation (i.e. two sessions instead of one) and less of chemotherapy (5 mg kg$^{-1}$ *vs* 10 mg kg$^{-1}$) and be challenged to a 'drug-alone' schedule inducing a high remission rate and at least a doubling of the survival time. Under such conditions, it has been demonstrated that cavitation potentiated FUra and CDDP on both the clinical outcome in the mid-term and the survival (for FUra) in the long term. While 100% remissions were obtained with certain therapeutic schedules at day 30, some animals treated with the same schedules still died within 5 months of follow-up. This can be explained by the fact that cavitation, as well as FUra, induces a blockade of the cell cycle and an accumulation of cells in $G_0/G_1$ (Prat *et al.*, personal communication; Valeriote & Santelli, 1984); we thus suggest that some cancer cells, still present in the peritoneum at day 30, may re-enter the cell-cycle or continue tumour growth at a slower rate.

The survival curves show that in no case but the combination of high dose FUra with cavitation was a survival observed over 5 months after tumour induction. In the case of cavitation, this may be interpreted as a result of an incomplete irradiation which did not involve the whole abdominal cavity, and subsequently allowed for some remnant cells to progress to large tumours. Nevertheless, the combination of high dose FUra with cavitation is a potentially curative option, associated with a toxicity which is probably the consequence of an intestinal impairment.

This contrasts with the relatively low incidence of toxicity directly related to cavitation in all other groups: no animal died post-operatively, the growth curve of the rats was not significantly affected and most importantly, no toxic effect was noted on the morphology and kinetics of the intestinal epithelium 30 days after therapy.

Corroborated by the most recent results from other laboratories on the anti-tumour effects of cavitation and shockwaves (Oosterhof *et al.*, 1991), we now consider cavitation as a potential novel physical method for the treatment of some cancers, probably in combination with systemic therapies. However, the technique that we used in this study to produce cavitation is still very rudimentary and is not applicable to the man at its present stage of development. Further improvements are needed to: (1) either enhance the focusing of cavitation to obtain a selective tissue ablation in a well-defined target or, on the contrary, to de-focus the acoustic beam to achieve a large-field irradiation; (2) get rid of the infusion of gas bubbles whose pharmacokinetics, biodistribution and toxicity are not well understood, and produce cavitation through purpose-built generators.

We thank Mr B. Chauffert from INSERM U252, Dijon, for methodological advice and suggestions. Supported in part by the Région Rhône-Alpes and an ARC grant, No. 6833.

### References

CALABRESI, P. & CHABNER, B.A. (1990). Chemotherapy of neoplastic diseases. In *The Pharmacological Basis of Therapeutics*, Goodman and Gilman's (ed.) eighth edition, pp. 1201–1263. Pergamon Press: New York.

CHURCH, C.C. & MILLER, M.W. (1983). The kinetics and mechanics of ultrasonically-induced cell lysis produced by non-trapped bubbles in a rotating culture tube. *Ultrasound Med. Biol.*, **9**, 385–393.

DEAR, J.P., FIELD, J.E. & WALTON, A.J. (1988). Gas compression and jet formation in cavities collapsed by a shock wave. *Nature*, **332**, 505–508.

DELIUS, M., MULLER, M., VOGEL, A. & BRENDEL, W. (1989). Shock waves and cavitation. In *Biliary Lithotripsy*, Ferrucci, J.T., Delius, M. & Burhenne, H.J. (eds) pp. 23–28. Year Book Medical Publishers: Chicago.

FLYNN, H.G. (1964). Physics of acoustic cavitation in liquids. In *Physical Acoustics: Principles and Methods. Vol 1B*, Mason, W.P. (ed.) pp. 57–112. Academic Press: New York.

GAMBIHLER, S. & DELIUS, M. (1992). *In vitro* interaction of lithotripter shock waves and cytotoxic drugs. *Br. J. Cancer*, 66, 69–73.

GASTROINTESTINAL TUMOR STUDY GROUP (1984). Adjuvant therapy of colon cancer -Results of a prospectively randomized trial. *N. Engl. J. Med.*, **310**, 737–743.

MILLER, D.L. (1987). A review of the ultransonic bioeffects of microsonation, gas-body activation, and related cavitation-like phenomena. *Ultrasound Med. Biol.*, **13**, 443–470.

MILLER, D.L., THOMAS, R.M. & FRAZIER, M.E. (1991). Single strand breaks in CHO cell DNA induced by ultrasonic cavitation *in vitro*. *Ultrasound Med. Biol.*, **17**, 401–406.

NAKAMURA, S., YOKOI, Y., SUZUKI, S., BABA, S., MURO, H. (1992). Results of extensive surgery for liver metastases in colorectal carcinoma. *Br. J. Cancer*, **79**, 35–38.

OOSTERHOF, G.O.N., SMITS, G.A.H.J., DE RUYTER, A.E., SCHALKEN, J.A. & DEBRUYNE, F.M.J. (1991). Effects of high energy shock waves combined with biological response modifiers in different human kidney cancer xenografts. *Ultrasound Med. Biol.*, **17**, 391–399.

PRAT, F., PONCHON, T., BERGER, F., CHAPELON, J.Y., GAGNON, P. & CATHIGNOL, D. (1991*a*). Hepatic lesions in the rabbit induced by acoustic cavitation. *Gastroenterology*, **100**, 1345–1350.

PRAT, F., CHAPELON, J.Y., CHAUFFERT, B., PONCHON, T. & CATHIGNOL, D. (1991*b*). Cytotoxic effects of acoustic cavitation on HT-29 cells and a rat peritoneal carcinomatosis *in vitro*. *Cancer Res.*, **51**, 3024–3029.

REGISTRY OF HEPATIC METASTASES (1988). Resection of the liver for colorectal carcinoma metastases: a multi-institutional study of indications for resection. *Surgery*, **103**, 278–288.

RUSSO, P., STEPHENSON, R.A., MIES, C., HURYK, R., HESTON, W.D.W., MELAMED, M.R. & FAIR, W.R. (1986). High energy shock waves suppress tumor growth *in vitro* and *in vivo*. *J. Urol.*, **135**, 626–628.

RUSSO, P., MIES, C., HURYK, R.K., HESTON, W.D.W. & FIAR, W.R. (1987). Histopathologic and ultrastructural correlates of tumor growth suppression by high energy shock waves. *J. Urol.*, **137**, 338–341.

VALERIOTE, F. & SANTELLI, G. (1984). 5-Fluorouracil (FUra). *Pharmac. Ther.*, **24**, 107–132.

WARLTERS, A., MORRIS, D.L., CAMERON-STRANGE, A. & LYNCH, W. (1992). Effect of electrohydraulic and extracorporeal shock waves on gastrointestinal cancer cells and their response to cytotoxic agents. *Gut*, **33**, 791–793.